# Water retention influences thigh skin temperature variation post-exercise: preliminary study of bioimpedance analysis and thermography data

**DOI:** 10.3389/fspor.2025.1516570

**Published:** 2025-02-14

**Authors:** Alessandra Amato, Luca Petrigna, Martina Sortino, Paulo Roberto S. Amorim, Giuseppe Musumeci

**Affiliations:** ^1^ Department of Biomedical and Biotechnological Sciences, Section of Anatomy, Histology and Movement Science, School of Medicine, University of Catania, Catania, Italy; ^2^ Department of Physical Education, Federal University of Viçosa, Viçosa, Minas Gerais, Brazil; ^3^ Research Center on Motor Activities (CRAM), University of Catania, Catania, Italy

**Keywords:** body composition, exercise, body temperature regulation, muscle, thermography, athletic performance

## Abstract

**Objectives:**

This study aimed to investigate the influence of body composition variables, focusing on the extracellular water level and gender difference, on infrared thermography detection during and post exercise.

**Method:**

One hundred two participants were included in the study. Body composition was analyzed by bioimpedance, and three thermal imaging were taken before, at the end, and 5 min after a vigorous exercise. First, participants were divided by gender, and differences in skin temperature variation during exercise were highlighted. In the second analysis, the subjects were divided into three groups depending on the percentage of extracellular water. The correlation between body composition variables and skin temperature at the 3-time points was studied.

**Results:**

an association between extracellular water (%) and basal thigh temperature both in the dominant leg (r: −0.27, *p* < 0.01) and non-dominant leg (r: −0.26, *p* < 0.01) was found; temperature variation analysis shows a significative temperature reduction between baseline and the end of exercise in both leg for (non-dominant: *p* < 0.001; dominant: *p* < 0.001) and a significative skin temperature increase after 5 min recovery, 0.14°C for the dominant leg (*p* > 0.05) and 0.12°C for the non-dominant leg (*p* > 0.05) considering the whole group. However, when we considered the separate group for extracellular contente the same significative decrease was found just in the lower water retention group (*p* < 0.05) and medium water retention group (*p* < 0.05). The high water retention group showed an opposite skin temperature trend in 5-min post-exercise recovery and had lower skin temperature at each time point compared with the other groups. The female group had lower skin temperature than the male at each time point.

**Conclusion:**

Water retention could influence basal skin temperature and the temperature variation following vigorous exercise. A value of less than 45% of extracellular water should be considered for reliable use of thermal imaging. Further studies are needed to confirm this value.

## Highlights

• Extracellular cellular water and thigh skin temperature are associated
• Extracellular water >45% showed an opposite skin temperature trend during the recovery phase compared with the normal skin temperature trend during exercise
• Extracellular water >45% has lower skin temperature than normal value at each time point
• site-specific association between leg fat mass and thigh skin temperature is identified for the first time.

## Introduction

In recent years, the literature on Infrared thermography (IRT) on the human body has increased because of its applications in several fields. In clinical medicine, IRT is engaged in postural (1), joint inflammation, degenerative pathologies, diabetes, breast cancer, dentistry, and cardiovascular system evaluation (2–5), evaluation. In sports science, through the detection of temperature asymmetries, IRT is useful in injury prevention by detecting inflammation of underlying muscle groups (6, 7).

Furthermore, in this field, IRT can provide information on muscle metabolism and vascular conditions (8). The interest behind this tool is that it is contactless, radiation-free, non-invasive, and inexpensive (9, 10). This tool detects body heat radiation (11), allowing the evaluation of the skin temperature in a pre-determined region of the body, the region of interest (ROI), through a summation of heat expression from the deeper tissues to the upper levels and blood flow (12). However, different factors could influence the IRT procedure, as a recent scoping review highlighted (2).

Standardization interests the data acquisition and processing and the creation of the region of interest (6) thus, standard operating procedure can help in this regard (13). The environmental conditions and settings, equipment accuracy, and physiological factors are some examples of counting factors (14). To minimize most of the confounding factors, the checklist entitled “Thermographic Imaging in Sports and Exercise Medicine (TISEM)” (15) was proposed. On the other side, factors such as the thickness or composition of the tissue (14) or other characteristics of the participants could also influence temperature detection. Body composition is being studied in the recent literature as one of them. However, it is unclear to date which body composition characteristics, in particular, should be attended to before performing IRT to have accurate and reliable results. According to different studies, there is a negative correlation between the total body fat percentage and the skin temperature evaluated through IRT (16).

However, just the total fat mass was evaluated as a possible body composition variable that affected the IRT. In addition, the tools used in these studies were error-prone and not discriminating between body structures, or composition, such as body mass index (BMI) (17), or expensive and not easy to find, especially in the sports context where IRT is more widely used, such as dual-energy x-ray absorptiometry or computed tomography scan (18, 19).

Another variable to date not investigated that could be an important variable to consider during IRT is the percentage of total body water (TBW). The TBW compartment consists of the extracellular water (ECW) and the intracellular water (ICW). The ratio ECW/TBW gives us information on cellular hydration.

ECW is considered to be related to dehydration; excessive fluid retention is known to cause increased morbidity (20). Normal values of the ECW/TBW ratio are about 0.38–0.40 (40%), values of up to 45% of TBW are tolerable, and ECW levels above about 45% of TB indicate water retention (21–24).

The bioelectrical impedance analysis (BIA) is an indirect, quick, and non-invasive method used for body composition analysis by measuring the resistance and reactance of biological tissue at multifrequency electrical currents. It is an accurate and cheaper tool that could be used in different health conditions (25). Individual tissues have peculiar electrical conduction properties. Resistance indicates the conductive characteristics of bodies and fluids; it decreases with increasing proportions of water in tissues. Reactance is related to the volume of ICW, indicating the capacity of the cell membrane, it is the opposition of the capacitor to alternating current. These two electrical values are used in the equations to calculate the electrical properties of tissues in the various BIA models (26).

The BIA is highly correlated with DXA (17, 27) and has been validated as an alternative to the subjective and biochemical indicators of hydration status (28, 29).

Water, along with the electrolytes dissolved in it, is particularly important in conducting electricity through the body. BIA could esteem TBW with an error of 1.5–2.5 kg by passing a small electrical current (50 kHz) through the body and measuring the impedance to that current (30). After estimating the TBW through established equations and models, the BIA estimates the volume of extracellular and intracellular fluids (31). All BIA models are based on the assumption that the resistance at 50 kHz is proportional to the TBW and from the TBW is distinguished, by subtraction, the resistances of ECW are richer in chloride, and the resistances of ICW are richer in more potassium. Thus, the estimation of TBW, ECW, and ICW is based on regression models taking into account the current passage of 50 kHz in which the formula is adjusted by population-derived indices (age, sex, height.) to reduce interindividual errors (26). Thus, the reference method to assess TBW, the deuterium oxide (D2O), is used to calibrate the various BIA models and make it possible to calculate impedance (30).

Adipose tissue and extracellular water have active electrical resistance. In contrast, reactance arises at the cell membrane of tissue with high water content, which acts like a capacitor composed of two covers and a dielectric layer.

Just one study (19) adopted BIA to associate body variables with thermal camera parameters. However, this study (19) considered the correlation just of the total body fat percentage (with no segmental limbs analysis) and the skin temperature variation after exercise in the upper limb, and only in male participants. Therefore, considering the current shortcomings in the literature, the study aimed to investigate the potential influence of body composition variables on thermal imaging detection after vigorous exercise by focusing on water retention and gender differences. This could allow us to understand whether IRT can be used as an effective tool to monitor the performance of participants with different physical characteristics.

Eventually, the study also wants to provide a value above which the thermal image loses effectiveness in this condition. For the first time, this study analyzed the influence of ECW and segmental body fat, assessed by BIA, on skin temperature variation during exercise in one of the anatomical segments most prone to fat accumulation, the thigh.

## Methods

### Participants

Participants were recruited from students of Sport Sciences at the University of Catania (Italy). They were included if they were 18 years old, injury-free, and able to perform a vigorous physical test. They were excluded if they presented dysmetabolism, eating disorders, lower limb injuries in the previous six months, disease of the locomotor system, or used not removable electronic devices. Pregnant women were excluded. A self-report questionnaire was administered to collect personal information: health status, frequency intensity time and type of structured physical activity performed during the week, and eating habits. In addition, the last section of the questionnaire included the “Waterloo Footedness Questionnaire” (32) to identify the dominant leg.

The study was designed and approved by the Research Center on Motor Activities (CRAM) Scientific Committee (Protocol n.: CRAM-020-2021, 20/12/2021). Participants provided voluntary informed consent, aligning with international bioethical standards as per the Declaration of Helsinki. Before the study, the protocols of the study, the risks, and the benefits of the study were presented to the participants. Participants did not receive payment and could withdraw from the study at any time Participants provided their written consent before the start of the study. Participants also permitted their data to be used anonymously.

### Instruments

The study adopted a thermal infrared camera, a multifrequency bioimpedance analyzer, and a plicometer [GIMA- Gessate (MI), Italy].

The infrared thermos camera adopted was the FLIR E54 camera (Wilsonville, OR, USA) with a 320 × 240 pixels detector resolution and thermal sensitivity <0.04°C. Its validity and reliability are presented in the introduction.

A bioelectric bioimpedance method through the body composition analyzer Tanita (MC-780A; TANITA Corporation, Tokyo, Japan) has been adopted to study body composition. The instrument has eight electrode multi-frequency (5 kHz/50 kHz/250 kHz) segmental and predicts the body mass characteristic from resistance and reactance of a constant current source (∼90 A). It wants to measure the resistance of individual tissues to the electrical impulse. Adipose tissue and extracellular water present active electrical resistance. This technology presents validity and reliability (33). This instrument has the certificate 93/42 EEC (EU norm for medical devices). The Polar. OH1 optical HR sensor from Polar. (Polar Electro Inc., Bethpage, NY, USA) was used to measure exercise intensity by heart rate.

### Experimental design

During the first phase, the BIA and the first IRT at baseline (T_base_) were collected. A second IRT (T_exe_) was proposed immediately after a 30-s vigorous exercise. The last IRT (T_rest_) was prosed after 5 min rest from the end of the exercise. At the end of the evaluation, thigh skinfold thickness was also measured in both legs.

### Thermal imagine protocol

The IRT acquisitions were performed according to the TISEM checklist (15). A tripod was placed in a room with a set temperature between 22° and 24° and humidity of 50%, 1.5 m away from the participants, to standardize the image acquisition; emissivity was set at 0.98 (34). Participants were asked to wear shorts to not influence the leg temperature; moreover, participants rested for 15 min in the evaluation room before the thermogram acquisition for acclimatization (35). During the imaging, participants stood upright with their legs to the camera, arms by their sides, facing the camera. The thermograms of both the dominant and non-dominant leg were acquired.

**Figure 1:**
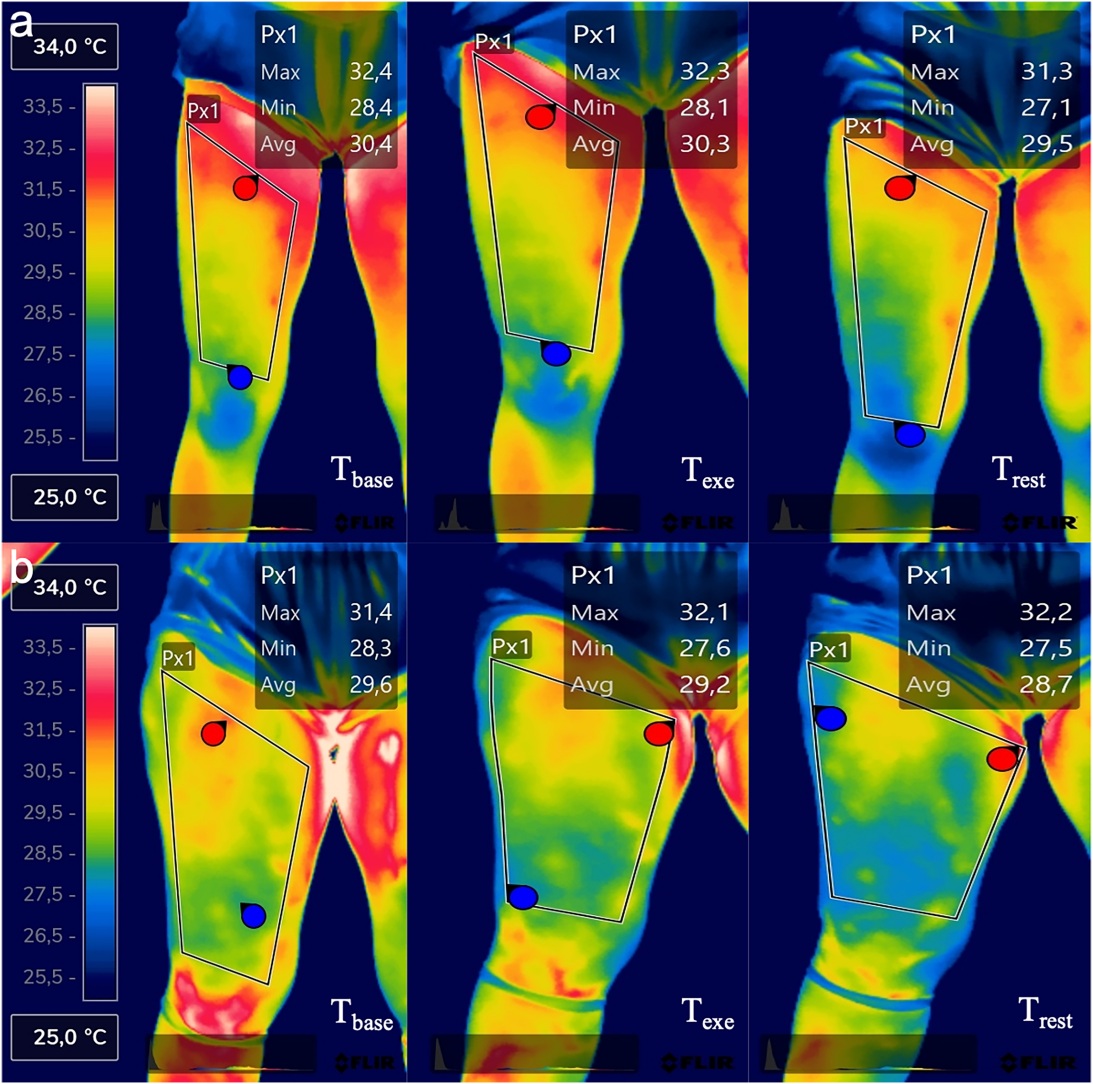
Thermal imaging of one representative participant of the LWR group **(a)** and one representative participant of the HWR group **(b)** in the three-time evaluation points.

Using the FLIR Thermal Studio PRO software, version number 1.9.38.0, the thermograms were analyzed. Two specular regions of interest (ROI) were created. One for the dominant and one for the non-dominant thigh. The ROI was drawn with a polygonal shape, having as the lower margin the patella (excluded), as the upper margin the groin line, and the lateral margins were drawn considering the line as close as possible to the thigh margin without going to the outer background. The center of the ROI coincided with the point where the plica was detected. Figure 1 helps to better understand the ROI selection and the temperature difference in the three-time evaluation.

### BIA protocol

Participants were instructed not to have breakfast before the BIA nor to drink coffee or other drinks. The evaluation was performed early in the morning, between 8 and 9 am. No heavy exercise had to be performed or alcohol intake in the previous 12 h.

Participants were barefoot, wearing shorts and tops. They were instructed to remove metals such as rings, bracelets, or necklaces. Then, participants were invited to step onto the bioimpedance analyzer scale and stand in a standing position. After the body weight evaluation, they were instructed to hold the two handles with arms separated from the other body segments and with the legs slightly apart (28). The variables extrapolated from TANITA PRO software 2.0 for this study were the total body fat (FAT %), TBW%, ECW %, dominant leg fat (%), and non-dominant leg fat (%), BMI (kg·m^−2^), and body weight (kg). All data are provided automatically in a Microsoft Excel® (Microsoft Corp., Redmond, WA) spreadsheet.

### Thigh skinfold thickness evaluation

The evaluation was performed with a skinfold caliper [GIMA- Gessate (MI), Italy] (36). The plica considered was at the thigh. The same evaluator performed all the measurements. During the measurements, participants stood with the leg relaxed (with the weight of the body resting on the contralateral limb) and the skin dry; the exact point of measurement is established using the tape measure by measuring the half distance between the iliac crest and the center of the knee as reference points. Plicae are obtained by taking the tissues between the thumb and forefinger 1 cm away from the plicometer and placing them on the point to be measured (Slim Guide, Creative Health Products, Ann Arbor, Michigan, USA) (37).

### Physical activity protocol

The test adopted is the 30-s countermovement jump. The participant had to perform as many squat jumps as possible in 30 s. According to the literature, 30 s is an adequate duration to induce ATP-PC and maximal glycolytic power (38). This reliable evaluation followed the procedure previously suggested (39): participants had to keep the trunk vertically. Differently from the protocol where the akimbo position was required, participants could use their upper limbs. The idea to propose the test with the arm swing is because this exercise aimed to reach the highest physical effort possible in 30 s free body and with a lower limb target exercise. According to the literature, the performance of a jump test with an arms swing increases the work done by the participant (40, 41). Participants had to flex the knee at about 90° in the transition between the eccentric-concentric phases Participants were encouraged to do the best possible performance to win the challenge against their peers. Heart rate was measured during the tests to assess exercise intensities.

### Statistical analysis

Statistical tests were run using SPSS Statistics 29 software programs. A significance level of 5% (*p* < 0.05) was used. The normality of data was analyzed with Shapiro–Wilk's test. For this reason, non-parametric statistical tests were used for the variables with no normal data distribution. Spearman's Rho was used to test the correlation between skin temperature variables and body composition variables, considering both the total body and the single-leg parameters. In addition, the maximal heart rate for each subject was calculated by using the formula HR = 220- age, and then by the peak heart rate reached during exercise, the percentage of maximal heart rate was calculated and used to identify the exercise intensity variable. The correlation between the intensity of exercise with the skin temperature variables and body composition variables was calculated with Spearman's Rho. After the normality check, the Mann–Whitney test and the Student *t*-test were used to investigate the gender groups in body composition variables.

Repeated measures ANOVA was used to detect average temperature variation between baseline (T_base_), post-exercise (T_exe_), and after 5 min to the end of exercise (T_rest_) in the whole group and between gender groups adjusted for multiple comparisons with Bonferroni.

In a secondary analysis, the participants were divided into three groups based on the percentage of extracellular water. Values of extracellular water (%) were assessed and used for separating the participants into low water retention (LWR) values from 38% to 40% of ECW, medium water retention (MWR) values from 41% to 44% of ECW, and high water retention (HWR) value from 45% to 48% of ECW. According to the ECW normal value described above, the LWR group had normal values of ECW, MWR had values considered tolerable of ECW, and HWR had values considered above the average of ECW thus, these participants had water retention (24).

Finally, Repeated measures ANOVA was performed by considering the within and between (LWR, MWR, HWR) groups factors to understand if the percentage of extracellular water could influence the evaluation of average temperature variation from the baseline to immediately after exercise and after 5 min of rest after exercise, (Figure 1). Values are expressed as mean ± standard deviation.

## Results

One hundred two participants were included in the data analysis [22.75 ± 5.05 years old, BMI 23.11 ± 3.65 (kg·m^−2^), Body Fat 19.48 ± 7.61 (%)]. Sex distribution was 58,8% male (n°60) and 41,2% female (n°42). A statistically significant difference was found between the gender groups in all body composition variables: body fat (%) (male: 15.63 ± 4.4, female: 24.98 ± 7.9; *p* < 0.001), total body water (%) (male: 59.91 ± 3, female: 51.66 ± 5.4; *p* < 0.001), extracellular water (%) (male: 40.94 ± 0.98, female: 43.44 ± 2.6; *p* < 0.001), dominant leg fat % (male: 13.27 ± 3.3, female: 32.30 ± 6.3; *p* < 0.001), and non-dominant leg fat % (male: 13.66 ± 3.6, female: 32.50 ± 6.2; *p* < 0.001).

### Correlation analysis

All the dependent variables were non-normally distributed (Shapiro–Wilk's test *p* < 0.05). Spearman's Rho was used. The intensity of exercise (% of Max Heart Rate) was not correlated with any of the variables of thermography and body composition: dominant and non-dominant leg temperatures (°C) (*p* > 0.05), TBW (%) (*p* > 0.05), ECW(%) (*p* > 0.05), Body fat (%) (*p* > 0.05). The body fat (%) of the whole sample was correlated negatively with TBW (%) (r: −0.86; *p* < 0.001) and positively with ECW (%) (r: 0.46 *p* < 0.001). However, no correlation was found between BMI (kg·m^−2^) or weight (Kg) and ECW (*p* > 0.05); TBW (%) was negatively correlated with ECW (%) (r: −0.66 *p* < 0.001).

In both legs, there are positive correlations between the temperature at baseline and Total body water (%); instead, negative correlations were found between the basal temperature of each leg and Extracellular water (%), body fat (%), leg fat mass (%), and thigh skinfold thickness (mm). Table 1 details the correlation analysis between the average basal temperature of both legs and each body composition variable.

**Table 1:** Correlation analysis between skin average temperature at baseline for each leg and body composition variables.

		TBW (%)	ECW (%)	Body fat (%)	Dominant thigh skinfold thickness (mm)	Dominant leg fat (%)
Dominant leg basal T_avg_	Spearman's rho	0.54^b^	−0.27^b^	−0.54^b^	−0.52^b^	−0.55^b^
	Sig. (2-tailed)	<0.001	0.006	<0.001	<0.001	<0.001
		TBW (%)	ECW (%)	Body fat (%)	Non-dominant thigh skinfold thickness (mm)	Non-dominant fat (%)
Non-dominant leg basal T_avg_	Spearman's rho	0.51^a^	−0.26^a^	−0.50^b^	−0.51^b^	−0.53^b^
	Sig. (2-tailed)	<0.001	≤ 0.01	<0.001	<0.001	<0.001

TBW, total body water; ICW, intracellular water; ECW, extracellular water; T_avg_, average temperature.

^a^ Correlation is significant at the 0.05 level (2-tailed).

^b^ Correlation is significant at the 0.01 level (2-tailed).

Particularly, extracellular water (%) was negatively correlated with dominant leg basal temperature (r: −0.21; *p* < 0.05) and non-dominant leg basal temperature (r: −0.26; *p* ≤ 0.01). Segmental leg fat mass was negatively correlated with leg basal temperature for the dominant (r: −0.55; *p* < 0.001) and non-dominant (r: −0.53; *p* < 0.001) (Table 1).

No correlation was found between the average thigh temperature at baseline in both legs and the body weight (*p* > 0.05).

### Temperature variation analysis

#### Whole sample temperature variation

The results of the ANOVA repeated measures performed considering the whole sample showed a mean difference decrease of 0.52°C for the dominant leg (*p* < 0.001) and of 0.63°C for the non-dominant leg (*p* < 0.001) between T_base_ and T_exe_. After 5 min from the end of the exercise, there was an increase of 0.14°C (dominant leg, *p* > 0.05) and 0.12°C (non-dominant leg, *p* > 0.05).

#### Gender group temperature variation

**Figure 2:**
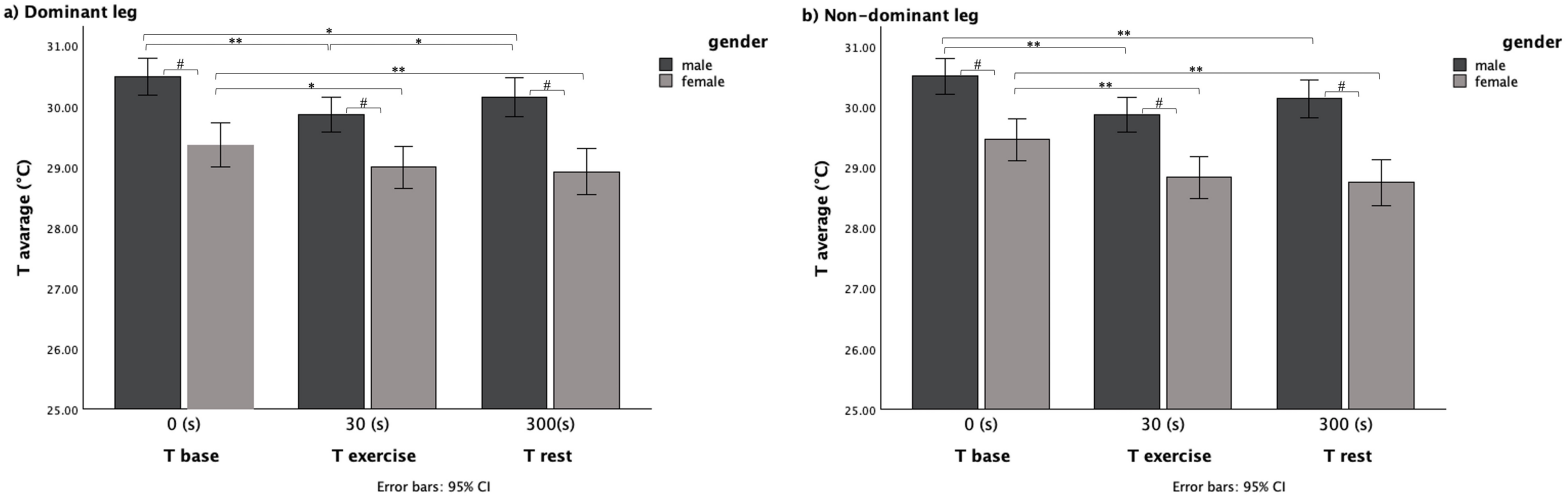
Average temperature variation within the three time evaluation and between gender for **(a)** dominant leg and **(b)** non-dominant leg.

ANOVA repeated measures performed following gender group distribution show that the average temperature of both quadriceps significantly decreased between baseline and the end of exercise in both legs and both males and females; male group: dominant leg [T_base_ (°C) 30.5 ± 1.01, T_exe_ (°C) 29.86 ± 1.11; *p* < 0.001]; non-dominant leg [T_base_ (°C) 30.5 ± 1.01, T_exe_ (°C) 29.88 ± 1.10; *p* < 0.001], (Figure 2); female group: dominant leg [T_base_ (°C) 29.35 ± 1.42, T_exe_ (°C) 29.00 ± 1.15; *p* < 0.05]; Non-dominant leg [T_base_ (°C) 29.46 ± 1.31, T_exe_ (°C) 28.84 ± 1.16; *p* < 0.001], (Figure 2).

Same significantly lower temperature than baseline temperature was maintained after 5 min to the end of exercise in both legs and in both sexes; male group: dominant leg [T_base_ (°C) 30.5 ± 1.01, T_rest_ (°C) 30.15 ± 1.14; *p* < 0.05]; non-dominant leg [T_base_ (°C) 30.5 ± 1.01, T_rest_ (°C) 30.14 ± 1.10; *p* < 0.01], (Figure 2); female group: dominant leg [T_base_ (°C) 29.35 ± 1.42, T_rest_ (°C) 28.92 ± 1.41; *p* < 0.01]; non-dominant leg [T_base_ (°C) 29.46 ± 1.31, T_rest_ (°C) 28.75 ± 1.40; *p* < 0.001], (Figure 2).

However, there was a statistically significant increase in temperature between T_exe_ and T_rest,_ only in the dominant leg of the male group [T_exe_ (°C) 29.86 ± 1.11, T_rest_ (°C) 30.15 ± 1.14; *p* > 0.05], (Figure 2).

Pairwise comparisons between gender groups showed a significant difference in each time point (*p* < 0.001) with the male group with the higher skin temperature (Figure 2).

#### ECW group temperature variation

**Figure 3:**
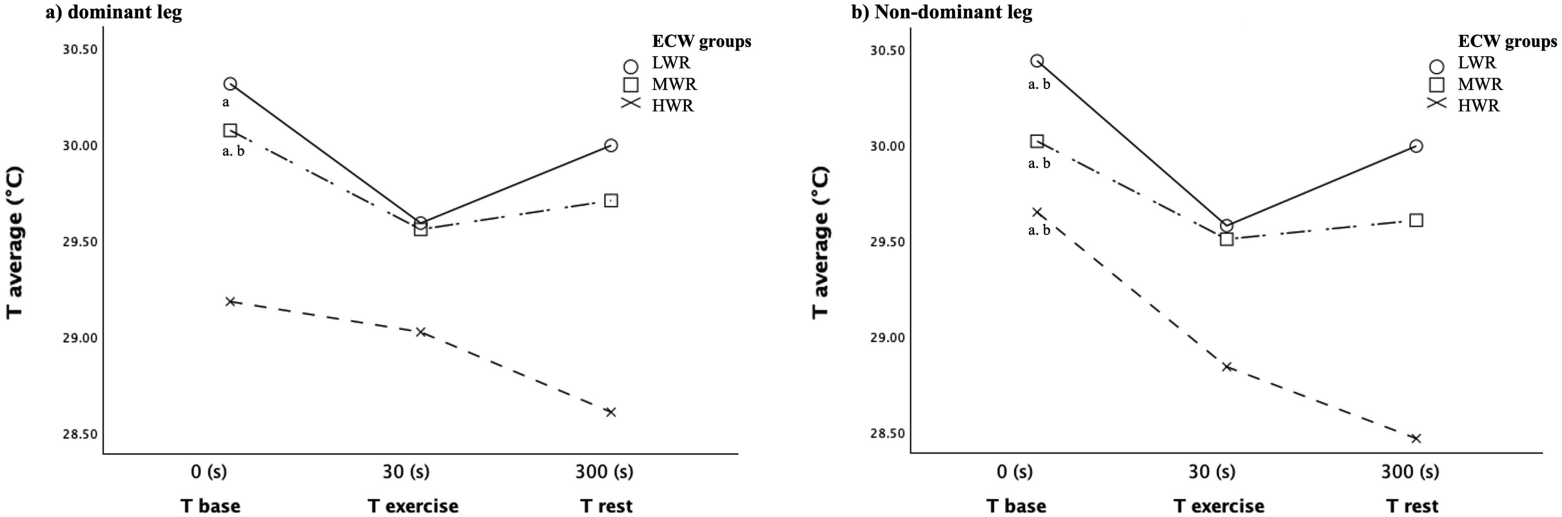
Average temperature variation in the three time for **(a)** dominant leg and **(b)** non-dominant leg between LWR, MWR and HWR groups.

Pairwise comparisons within groups show a significant temperature variation between T_base_ and T_exe_ in LWR and MWR groups for the dominant leg and in LWR, MWR, and HWR groups (*p* < 0.05) for the non-dominant (Figure 3). The main differences between T_base_ and T_exe_ show temperature decreases in all three groups.

A significative difference in temperature between T_base_ and T_rest_ was found just in the MWR group for the dominant leg (*p* < 0.05) and in all groups for the non-dominant legs (*p* < 0.05) (Figure 3). The mean difference showed that T_rest_ after 5 min to the end of the exercise was lower than the basal skin temperature in all ECW groups (Table 2).

**Table 2:** Pairwise comparisons of t_avg_ within LWR, MWR and HWR groups.

a) Dominant leg					b) Non-dominant leg				
Groups	(I) temperature	(J) temperature	Mean Difference (°C) (I–J)	Sig.^b^	Groups	(I) temperature	(J) temperature	Mean Difference (°C) (I–J)	Sig.^b^
LWR	T_base_	T_exe_	0.725^a^	<0.001	LWR	T_base_	T_exe_	0.862^a^	<0.001
		T_rest_	0.321	0.141			T_rest_	0.446^a^	.041
	T_exe_	T_base_	−0.725^a^	<0.001		T_exe_	T_base_	−0.862^a^	<0.001
		T_rest_	−0.404	0.075			T_rest_	−0.417	0.061
	T_rest_	T_base_	−0.321	0.141		T_rest_	T_base_	−0.446^a^	0.041
		T_exe_	0.404	0.075			T_exe_	0.417	0.061
MWR	T_base_	T_exe_	0.515^a^	<0.001	MWR	T_base_	T_exe_	0.512^a^	<0.001
		T_rest_	0.365^a^	0.006			T_rest_	0.414^a^	0.002
	T_exe_	T_base_	−0.515^a^	<0.001		T_exe_	T_base_	−0.512^a^	<0.001
		T_rest_	−0.150	0.270			T_rest_	−0.098	0.460
	T_rest_	T_base_	−0.365^a^	0.006		T_rest_	T_base_	−0.414^a^	0.002
		T_exe_	0.150	0.270			T_exe_	0.098	0.460
HWR	T_base_	T_exe_	0.158	0.572	HWR	T_base_	T_exe_	0.808^a^	0.005
		T_rest_	0.575	0.063			T_rest_	1.183^a^	<0.001
	T_exe_	T_base_	−0.158	0.572		T_exe_	T_base_	−0.808^a^	0.005
		T_rest_	0.417	0.192			T_rest_	0.375	0.232
	T_rest_	T_base_	−0.575	0.063		T_rest_	T_base_	−1.183^a^	<0.001
		T_exe_	−0.417	0.192			T_exe_	−0.375	0.232

Based on estimated marginal means.

^a^ The mean difference is significant at the .05 level.

^b^ Adjustment for multiple comparisons: Least Significant Difference (equivalent to no adjustments).

No significant temperature variation between T_exe_ and T_rest_ was found in all groups and for both legs (Figure 3). However, after 5 min from the end of the exercise, mean differences (Table 2) showed an increase of 0.41°C (dominant leg), 0.42°C (non-dominant leg) in the LWR group and 0.15°C (dominant leg), and 0.10°C (non-dominant) in MWR but there was a skin temperature decrease in the HWR group of 0.42°C for the dominant leg and 0.36°C for the non-dominant leg highlighting an opposite skin temperature trend in the HWR.

The mean differences between the three temperature evaluations for both legs are shown in Table 2.

Pairwise comparisons between ECW groups, considering the dominant leg, show at baseline evaluation significant differences between HWR and both LWR (*p* < 0.05) and MWR (*p* < 0.05) groups, but not between LWR and MWR groups. The HWR group skin temperature was 1.13° C lower than the LWR group and 0.89°C lower than the MWR group at the baseline. There are no statistically significant differences in average temperatures between the groups for both legs at the end of exercise (Texe). However, the HWR group skin temperature was 0.57°C lower than the LWR and 0.53°C lower than the MWR groups in the dominant leg, and 0.74°C lower than the LWR and 0.67°C lower than the MWR groups in the non-dominant leg.

After 5 min from the end of the exercise, there were significant differences between the HWR group and both LWR (*p* < 0.005) and MWR (*p* < 0.05), but not between LWR and MWR groups in both legs. Particularly, the HWR skin temperature was 1.39°C lower than the LWR group and 1.10°C lower than the MWR in the dominant leg (Table 3, a), and 1.53°C lower than the LWR (*p* < 0.05) and 1.14°C lower than the MWR Group in the dominant leg (Table 3, b). The HWR group had a lower temperature than the other two groups in all three time assessments, (Table 3, a and b).

**Table 3:** Pairwise comparisons of t_avg_ between LWR, MWR and HWR groups.

Dominant leg					Non-dominant leg				
Temperature	(I) Group	(J) Group	Mean Difference (°C) (I–J)	Sig.^b^	Temperature	(I) Group	(J) Group	Mean Difference (°C) (I–J)	Sig.^b^
T_base_	LWR	MWR	0.242	0.430	T_base_	LWR	MWR	0.420	0.158
		HWR	1.133^a^	0.014			HWR	0.792	0.074
	MWR	LWR	−0.242	0.430		MWR	LWR	−0.420	0.158
		HWR	0.891^a^	0.029			HWR	0.371	0.343
	HWR	LWR	−1.133^a^	0.014		HWR	LWR	−0.792	0.074
		MWR	−0.891^a^	0.029			MWR	−0.371	0.343
T_exe_	LWR	MWR	0.033	0.909	T_exe_	LWR	MWR	0.070	0.810
		HWR	0.567	0.183			HWR	0.738	0.091
	MWR	LWR	−0.033	0.909		MWR	LWR	−0.070	0.810
		HWR	0.534	0.158			HWR	0.667	0.085
	HWR	LWR	−0.567	0.183		HWR	LWR	−0.738	0.091
		MWR	−0.534	0.158			MWR	−0.667	0.085
T_rest_	LWR	MWR	0.287	0.374	T_rest_	LWR	MWR	0.388	0.231
		HWR	1.387^a^	0.004			HWR	1.529^a^	0.002
	MWR	LWR	−0.287	0.374		MWR	LWR	−0.388	0.231
		HWR	1.101^a^	0.011			HWR	1.141^a^	0.008
	HWR	LWR	−1.387^a^	0.004		HWR	LWR	−1.529^a^	0.002
		MWR	−1.101^a^	0.011			MWR	−1.141^a^	0.008

Based on estimated marginal means.

^a^ The mean difference is significant at the .05 level.

^b^ Adjustment for multiple comparisons: Least Significant Difference (equivalent to no adjustments).

## Discussion

The study aimed to investigate the influence of water retention and segmental fat mass on the skin temperature variation during and after vigorous exercise detected by IRT. A negative association between basal thigh skin temperature and lower limb segmental fat mass was found. The heat generated into deeper body tissues reaches the epidermis to promote thermoregulation, this occurs due to the temperature gradient and depends on the thermal conductivity of various body tissues (42), which depends on water content and affects body thermal conductivity. Fat tissue has low thermal conductivity. Therefore, the subcutaneous fat mass affects the heat emitted from the skin surface. This might justify our results with lower temperatures in regions with a higher fat percentage. We obtained the same results when we divided the group by gender, where the women's lower thigh temperature than men in all three-time points (*p* > 0.05) is explained by their higher limb fat %. Several studies found a similar negative association between total fat mass and segmental skin temperature (16, 18), adipose tissue acts as an insulating barrier, which influences heat modulation through the skin this alters the body's ability to respond to changes in environmental temperature at sites with excess adipose tissue (43). However, Salamunes et al. used the DEXA to evaluate the fat mass and considered only female participants (18); Reis et al. performed body composition analysis with DEXA considering just men. Neither study considered the influence of segmental body fat on the change in mean temperature during and post-exercise by also comparing males and females. Weigert et al. (19), in agreement with our results, showed that body fat and skin temperature have a negative correlation. However, he considered just the total body fat percentage, and he analyzed the temperature in the biceps brachii segments, which is not an excessive fat accumulation and retention point (44). In our study, the thigh was considered both for temperature and fat assessment and as a segment involved in exercise so for the first time segmental temperature has been associated with segmental fat of the limb direct involved in the exercise.

The thigh is considered among the points of greatest fat accumulation and water retention (44).

We found a negative correlation between ECW and the basal average temperature in both legs (Table 1) this could be justified by the characteristics of the water retention condition. Water retention is an excessive build-up of liquids, which generally accumulates in the circulatory system, body tissues, or specific cavities in the body, i.e., thighs (45). The retention is characterized by edema. This alteration was considered a component of the pathophysiology of adipose tissue diseases (46). However, there is not necessarily an association between retention and the amount of body fat, retention can have several causes also independent of fat accumulation (45). So it is reasonable to analyses the ECW variable independently from the fat % to understand it's influence on IRT. Extracellular fluid has altered resistance, the conductance of extracellular fluid is determined by the concentration of ions, with sodium being the most concentrated (47). Higher tissue sodium content results in lower impedance in legs affected by lipedema and is a major component of the composition of tissues affected by water retention. It's demonstrated that altered extracellular fluids contribute to the pathological skin characteristics of areas affected by lipedema (48) by altering the conductivity in these tissues and consequently the processes of skin thermoregulation (49). Skin temperature depends on the vascular supply of skin and subcutaneous tissues and the thermal conductivity of the skin. Areas affected by lipedema have been shown to have 35% lower blood flow than unaffected regions. Thermographic images of the participants with edema are characterized by inhomogeneous temperature with ischemic areas where the temperature is lowered (hypothermia) were the cold spots may be related to blood stasis (50). In our study the HWR group had a lower temperature than the other groups at all evaluation time points (Table 3). However, while T_base_ and T_rest_, this difference was significant, there was no difference between groups at the end of vigorous exercise (T_exe_).

Muscle region's thermal adaptation to exercise in our study showed a decreased temperature immediately after the exercise (T_exe_), both when we considered the whole group and when we considered separate groups for gender (Figure 2) or ECW content (Figure 3).

This temperature trend after exercise and rest time agrees with several articles that show that temperature tends to decrease during and at the end of exercise and increase during the recovery phase (51–54). Particularly, the temperature decrease seems to be about −0.4°C or more (51, 52). In our study, this range was complied in the LWR and MWR groups but not in the HWR (Table 2) that had an inhomogeneous trend between the dominant and non-dominant limb of 0.15°C and 0.80°C respectively underling that water retention could result in inhomogeneous temperature areas. The reduction in T_exe_ values in all groups could be explained by a redistribution of blood flow from the skin to the muscles activated during exercise to allow for the adequate supply of substances necessary for the increased muscle work. indeed, an indirect relationship between skin temperature during exercise and exercise intensity has been demonstrated (55). In our study, the vigorous intensity of the proposed exercise produced this effect, by reducing the temperature from T_base_ to T_exe_. Another mechanism that may contribute to a sudden decrease in skin temperature at the exercise start could be the evaporation of sweat that occurs due to the increase in metabolic activity during exercise, which causes the internal temperature to rise, producing sweat to reduce it at the skin level. However, in our study, this factor should be considered with caution, given the short duration of exercise. In addition, a relationship between sympathetic and parasympathetic activation and changes in skin temperature during physical exercise has been demonstrated (52). Usually, during the warm-up phase, there is a sympathetic neurogenic vasodilation, and during the cool-down phase, there is noradrenergic vasoconstriction in the muscles areas involded in exercise (56). Exercise influenced these events because it is related to several hemodynamic changes. Muscle activity results in an increase in body temperature due to the activation of the energy mechanisms involved (57), and this activates the cutaneous thermo-regulatory processes. Therefore, we have an altered skin temperature as a result of muscle activation during exercise. Thus, the decrease of skin temperature immediately following (T_exe_) the vigorous exercise we administered may be associated with skin vasoconstriction attributable to an increase in vasoconstrictor hormones released following the performance of all-out exercise (58).

In contrast, when we considered the temperature after the recovery phase (T_rest_), there was an increase in temperature in the whole sample, in the LWR and MWR groups but the temperature still decreased after 5-min recovery (T_rest_) in the HWR group. This condition was the same for the dominant and non-dominant limbs. Recent literature demonstrated that in normal conditions, the thermal images during recovery phases showed an increased cutaneous temperature because a new metabolic condition arises, requiring specific mechanisms for the release of the metabolic and mechanical heat produced during exercise.

During this period, the redistribution of blood flow follows an opposite direction from that immediately at the start of exercise, i.e., flow increases from the deeper tissues to the skin tissue inducing the activation of thermal dissipation processes (59):

Exercise produces an increase in blood nitric oxide (NO) concentrations, which is an important vasodilator of skin arterioles. NO promotes cutaneous vasodilation during the recovery phase in regions undergoing exercise by directly modulating nerve activity acting on smooth muscles (60). The result is an increased blood supply that causes an elevation of local temperature, which can be measured with thermal imaging cameras.

Other mechanisms could influence the skin temperature increase during the recovery phase such as glycogen resynthesis because the heat generated by these mechanisms occurs in the anatomical structures just under the skin (61), as well is important to consider the excess post-exercise oxygen consumption (EPOC), which varies directly according to the intensity and duration of the stimulus, which will influence skin temperature by the necessary dissipation of heat towards the state of body homeostasis. It has already been verified that the responses of IRTs in continuous (54) and progressive (53) exercise show different thermographsic responses.

However, If we look at the trend between T_exe_ and T_rest_ evaluation in the HWR group (Figures 3a,b), we have an opposite trend_._ A possible explanation is that HWR participants had ECW levels over the tolerable value of 45%, indicating retention, the subcutaneous tissue of areas characterized by edema resulting from retention is often ischemic areas with a lower temperature than the other regions of the same segment (Figure 1) where the subcutaneous circulation is impaired or altered blood stasis ineffective for the heat dissipation processes occurring during the recovery phase. However, further studies that would allow the investigation of subcutaneous tissue would be needed to accept this explanation. It is reasonable to assume that they may have slower local thermoregulation that cannot be detected in 5 min of recovery. Future studies should explore this relationship after different phases of recovery.

The importance of investigating how skin temperature reacts to exercise in participants with different characteristics could be linked to the accuracy of information we can derive about athletes and their thermal regulation by IRT (62). Because a winged subcutaneous tissue and circulation could give incorrect information regarding temperature symmetry between limbs. This element is an established method for injury prevention by quantifying training load (63) or the biomechanical imbalances between limbs (64) by IRT. Therefore, identifying a specific factor that could affect the detection of skin temperature by IRT could be a key point in the correct use of thermal imaging. In addition, identifying an ECW value above which the use of IRT may not be reliable would be crucial to having standardized protocols.

## Limitations and future studies

This study has some limitations. Participants were not divided according to the physical activity level; Future studies could stratify the participants according to the physical activity practiced or different characteristics of body composition. Only the acute effect of vigorous exercise protocol was proposed, in the future, the effect of other types of exercise should be studied. We focus only in the 5 min of recovery after the exercise, analysis of other recovery periods can add value to these initial results. Although all types of exercise are characterized by an increase in sympathetic activation and a subsequent decrease in parasympathetic activation, previous studies indicate high variability in IRT values before, after exercise, and during the recovery phase.

Therefore, it is difficult to establish segmental temperature values in the various phases of exercise to refer to. In addition, although the BIA agreed well with the reference method to assess TBW (the deuterium dilution) (65) the accuracy of predictive equations of BIA models is affected by several factors: the inhomogeneous nature of various body compartments, electrolyte balance independent of fluid changes, and large interindividual variation in differences in circumferences between various body segments. In addition, greater variation in total body volume may result in relatively smaller variations in body resistance and reactance that are below the level of BIA accuracy. However, a BIA segmental approach, as the tool used in our study, reduce BIA tools bias related to interindividual variability in body shape and cross-sectional areas. Future studies would also explore the influence of water retention on skin temperature variation in other body sites, at different times during exercise and during other recovery times to set a specific thermal imaging protocol.

## Conclusion

The present study identifies an association between segmental fat mass percentage and ECW with skin temperature, confirms the decrease in skin temperature at the beginning of exercise and its increase in the recovery phases; and, for the first time establishes that participants with ECW >45% exhibit an opposite temperature trend in 5-min post-exercise recovery in comparison with a normal trend. In employing thermal imaging as a tool for assessing post-exercise temperature, we should take into account the participant's water retention.

## Data Availability

The original contributions presented in the study are included in the article/Supplementary Material, further inquiries can be directed to the corresponding author.
